# A Novel Preparation of Special-Shaped Phosphors-in-Glass by Gel Casting with Isobam for High-Power WLEDs Lighting

**DOI:** 10.3390/ma15134667

**Published:** 2022-07-03

**Authors:** Hongmei Liu, Junjie Tian, Honghao Sun, Qian Xu, Jinyan Yu, Qing Yao

**Affiliations:** 1 School of Mechanical Engineering, Nantong University, Nantong 226019, China; liu.hm@ntu.edu.cn (H.L.); 2009310006@stmail.ntu.edu.cn (J.T.); yjinyanxiaoyu@163.com (J.Y.); 2 School of Transportation and Civil Engineering, Nantong University, Nantong 226019, China; 2018310004@stmail.ntu.edu.cn (H.S.); xuqian@ntu.edu.cn (Q.X.); 3 Xinglin College, Nantong University, Nantong 226019, China

**Keywords:** phosphors-in-glass, white light emitting diodes, gel casting, color rendering index, heat dissipation

## Abstract

Phosphors-in-glass (PiGs) regarded as a promising phosphor-converter for white light emitting diodes (WLEDs) is being researched widely. However, there are few reports on the effect of changing the shape of PiGs on the color rendering index (CRI) and heat dissipation of WLEDs. In this paper, gel casting with Isobam was first attempted in preparing special-shaped PiGs successfully. It exhibited that 76 wt.% was the optimum solid content based on the rheological properties of slurry and the shrinkage of green bodies. The sintering rate should be kept at a low speed and glass transition temperature (Tg) of glass powders must be higher than sublimation temperatures (Ts) of APS and Isobam. The CRI of PiGs was increased by about 27% after changing the shape of PiGs from cylinder to dome. Most importantly, operating temperature also reduced effectively the increase of the surface area of PiGs. Therefore, changing the shape of PiGs by gel casting with Isobam is a creative way for high-power WLEDs lighting.

## 1. Introduction

As the fourth-generation light source, white light diodes (WLEDs) have been widely used in the field of white lighting and backlight [1,2,3]. The combination of yellow Ce^3+^: Y_3_Al_5_O_12_ (Ce:YAG) phosphors and blue InGaN/GaN chip is the most common method to obtain WLEDs [4,5,6]. However, the further development of WLEDs is limited by the traditional phosphor-converter phosphors-in-silicones (PiSs) [7,8,9], because PiSs have problems such as thermal quenching of phosphors and degradation of resin at high temperature. Meanwhile, phosphors-in-glass (PiGs) with good heat dissipation and high heat resistance are a suitable phosphor-converter for high power WLEDs lighting [10,11,12].

At present, the research of PiGs often focus on three aspects: preparing novel phosphors particles [13,14,15], introducing ions into glass matrix [10,16,17] and reducing sintering temperature [18,19,20]. There are few reports on the effect of changing shape of PiGs on optical properties and heat dissipation. This is because the special shaped PiGs cannot be prepared by dry pressing and screen printing. However, it is obvious that the hemispherical dome-shaped structure can improve the performance of WLEDs [2,21,22]. Therefore, it is necessary to prepare specially shaped PiGs adopting a new forming method.

Gel casting is often used in the preparation of various high quality ceramic parts, especially for the complex ceramic parts [23,24,25]. The principle is that particles in the concentrated slurry are arranged by a three-dimensional network structure which is transformed from organic monomer [26,27]. Then, the slurry will be gradually solidified and formed. Among gel systems, a novel gel system based on a copolymer of isobutylene and maleic anhydride (Isobam) is concerned widely [28,29,30]. The advantages of this gel system are non-toxic, low addition, and easy to burn out. Hence, this provides a theoretical basis for the preparation of PiGs through gel casting with Isobam.

In this paper, it was the first time to prepare cylindrical and domed-shaped PiGs successfully adopting gel casting with Isobam. The rheological behaviors of concentrated slurry, shrinkage of green bodies, and different shapes on the heat dissipation performance of PiGs were systematically studied. In addition, the effects of different solid contents and shapes on optical properties of WLEDs were also analyzed. The combination of gel casting and PiGs not only broadened the application field of gel casting, but also promoted the further development of WLEDs in the field of high-power lighting.

## 2. Materials and Methods

**Figure 1:**
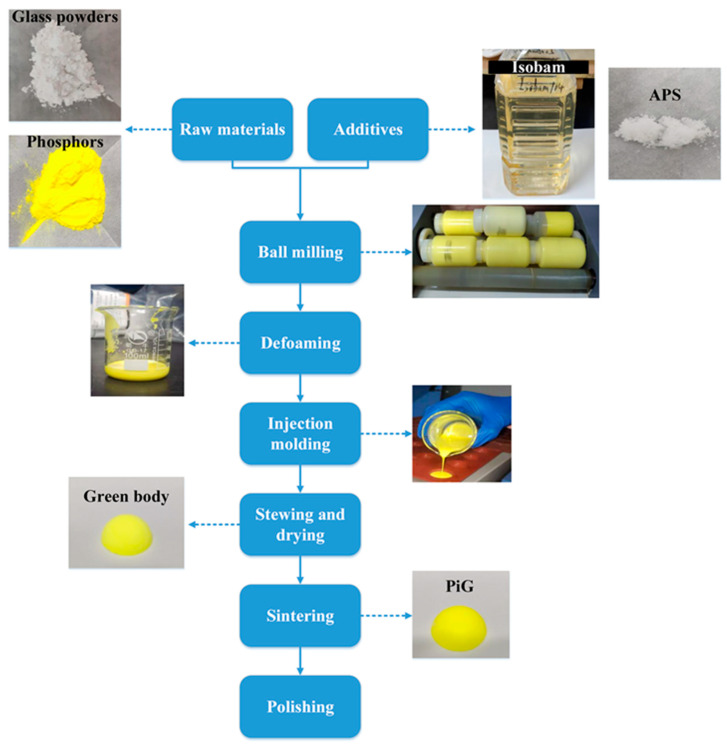
Process for preparing PiGs by gel casting with Isobam.

The preparation process for the PiGs using Isobam gel system was shown in Figure 1. First, the Ce:YAG phosphors (YAG-04, 99.99%, Shenzhen Looking Long Technology Co., Ltd., China) and glass powders (99.99%, Hebei Jiegui Mining Co., Ltd., China) were weighted according to table shown in Table 1 and mixed with deionized water by a rolling ball mill (GMS3-4, MITR, China) for 5 min. Second, 3 wt.% Isobam 104# (99.99%, Kuraray Co., Ltd., Osaka, Japan) and 1 wt.% (NH_4_)_2_S_2_O_8_ (APS, 99.99%, Aladdin, China) were added and continued to mix at 400 r/min for 1.5 h to get a slurry. Third, the slurry was casted into molds after vacuum degassing in a bubble-removing machine (HMV200, Shenzhen Hasai Technology Co., Ltd., China) and gradually gelled at room temperature for 24 h to obtain green bodies. Then, the green bodies were dried at 60 °C for 12 h in a drying oven (DHG-9053A, Shanghai Yiheng Technology Co., Ltd., China). Fifth, the green bodies were sintered at a sintering rate of 1 °C/min to 800 °C and kept for 30 min, and then cooled to room temperature in a muffle furnace (KSL-1100X-S, Hefei Kejing Material Technology Co., Ltd., China). Finally, the samples PiGs were polished based on different design requirements.

The glass transition temperature (Tg) of the glass powders, sublimation temperatures (Ts) of APS and Isobam were measured by differential scanning calorimetry (DSC 214Polyma, NETZSCH, Germany) with thermogravimetric analysis (TG) at a temperature rise rate of 10 °C/min. The rheological properties of PiGs slurry were analyzed by a parallel-plate rheometer (RheoWin MARS III, Haake, Germany) at a shear rate from 0.01 s^−1^ to 1000 s^−1^ at room temperature. The densities and shrinkages of PiGs before and after sintering were measured using the Archimedes method. The polished surfaces of PiGs and distribution of elements in PiGs were observed by scanning electron microscope (SEM, JSM-6510, JEOL, Kariya, Japan) with an energy dispersive spectrometer (EDS, Aztex, Oxford Instruments, Oxford, England) system. The distributions of phosphors in green bodies and PiGs were analyzed by using a confocal laser scanning microscope (CLSM, TCS SP5, Leica, Germany). The crystalline phases of phosphors, glass powders, Isobam, APS, green bodies, and PiGs were obtained by an X-ray diffraction system (XRD, X’Pert PRO MRD, PANalytical). The color rendering index (CRI) and color temperature of WLEDs were characterized in a 100 cm diameter integrating sphere using a photoelectric analysis system (HPCS6500, HOPOOCOLOR, China). The operating temperatures of WLEDs were obtained using a thermal infrared camera (MAG-F3/6, Shanghai Juge Technology Co., Ltd., China).

## 3. Results and Discussion

### 3.1. Sintering Process of Green Bodies

**Figure 2:**
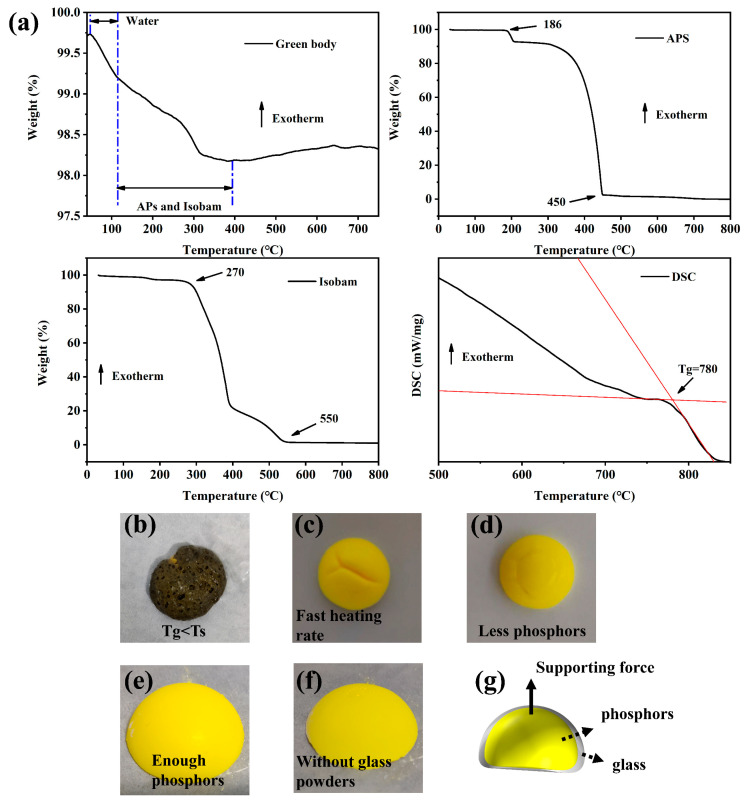
(**a**) Weight loss curves of green bodies, APS and Isobam and DSC curve of glass powders; (**b**) photos of PiGs with low Tg and (**c**) in fast sintering rate; (**d**–**f**) photos of PiGs with different phosphors content; (**g**) schematic diagram of phosphors supporting glass.

Figure 2a presents the whole process of weight loss of the green bodies of PiGs during sintering, Tg of the glass powders and Ts of APS and Isobam. Tg of the glass powders was 780 °C and Ts of APS and Isobam was about 186 °C and 270 °C respectively. Due to the evaporation of water, there was an obvious weight loss process before 100 °C [26]. The weight loss between 100 °C and 400 °C was due to the gradual elimination of Isobam and APS. This was in accordance with the Ts of Isobam and APS. After 400 °C, the weight of the green bodies did not decrease, which indicated that the organic matter in the PiGs has been removed. It was noteworthy that Tg of the glass powders must be greater than 400 °C. When the Tg is lower than 400 °C, it can affect the discharge of organic matter as shown in Figure 2b. The sintering rate also plays an important role in the preparation of PiGs. When the sintering rate is too fast, a depression displayed in Figure 2c occurs. It was attributed to that the three-dimensional network supporting the green bodies collapsed too fast. This implied that the sintering rate must be controlled slowly in the sintering process. Figure 2d–f exhibit different phosphors contents of PiGs after sintering at the temperature of 800 °C. The problem of PiGs with less phosphors was similar with that in Figure 2c but the shape of PiGs with enough phosphors and without glass powders kept intact. This implied that phosphors played a good role in keeping shapes of PiGs. As shown in Figure 2g, when the glass is in the molten state, the phosphors particles can provide a force to support the glass.

### 3.2. Physical Properties of PiGs

#### 3.2.1. Rheology Behavior

**Figure 3:**
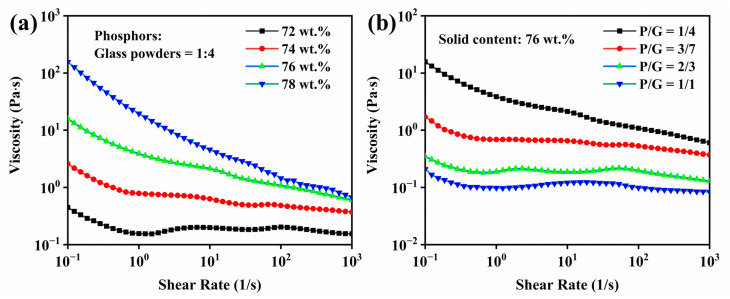
(**a**) Rheology properties of different solid contents in the same ratio and (**b**) different ratios in the same solid content.

The influences of solid contents and ratios on the viscosity of slurry with 3.0 wt.% Isobam and 1.0 wt.% APS is shown in Figure 3. With the increase of shear rate, all slurry exhibited an obvious shear-thinning behavior. Solid content was the main factor influencing the viscosity of slurry. As seen from Figure 3a, the viscosity of slurry increases when solid content increases from 72 wt.% to 78 wt.%. The viscosity of the slurry was too high to caste when solid content reached 78 wt.%. The changes of viscosities of slurry in the same solid content are displayed in Figure 3b. With the increase of phosphors and the decrease of glass powders, the viscosity decreased continuously. This was mainly due to the fact that the solubility of phosphors was greater than glass powders in water. In addition, it was noticeable that the viscosity of the slurry increases abnormally in some areas. It depended on the two main mechanisms. On the one hand, the agglomeration phenomenon led to viscous slurry [31]. On the other hand, when APS was added into the slurry, the electrostatic repulsion between particles would increase and then the viscosity decreased [30].

#### 3.2.2. Shrinkage Ratio and Density

**Figure 4:**
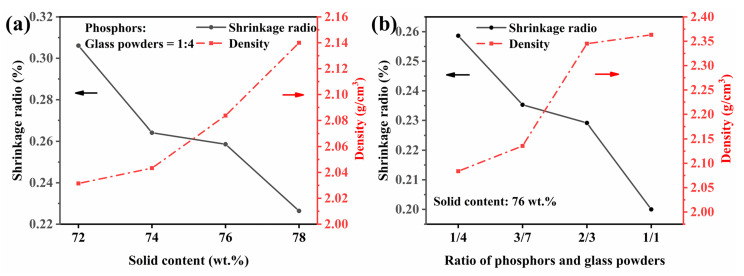
(**a**) The shrinkage ratios and densities of green bodies with different solid contents and (**b**) ratios of phosphors and glass powders.

Figure 4 exhibits the shrinkage ratios and densities of green bodies with different solid contents and ratios of phosphors and glass powders. It can be easily observed that the shrinkage ratio decreases as the solid content increases from 72 wt.% to 78 wt.% in Figure 4a. Obviously, this was owing to the fact that water content in green bodies was different under different solid contents [32]. As shown in Figure 2a, the remove of water was the main weight loss of the green bodies during the sintering process. The less water in green bodies of higher solid content resulted in lower shrinkages. Meanwhile, it can also be seen from Figure 4a that the density increases with the increase of solid content. It meant that high solid content was helpful to realize the near-net shaping of high-quality PiGs. Therefore, the optimal solid content is 76 wt.% after considering the shrinkage and rheological behavior. When phosphors increase and glass powders decrease at the same solid content, the shrinkage decreases as displayed in Figure 4b. The different sizes of phosphors particles and glass powders were the essential parameter governing the phenomenon. During the gelation process, the smaller particles were arranged more closely by three-dimensional network at the same solid content. It was also implied by the change of density shown in Figure 4b. A more important point was that changes in the density of PiGs may affect their optical properties.

### 3.3. Microscopic Characteristics of PiGs

**Figure 5:**
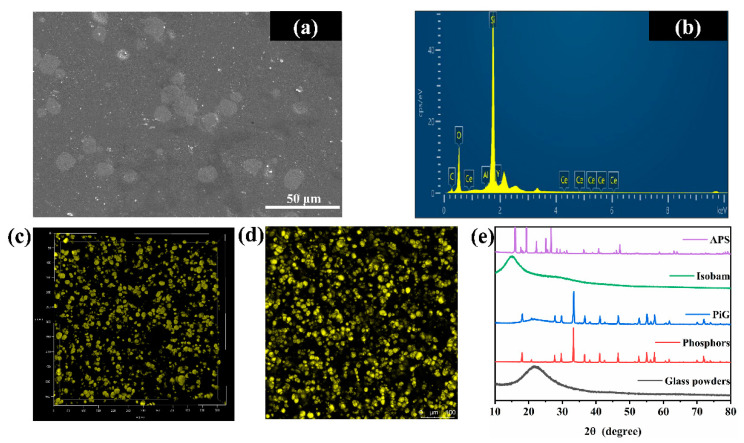
(**a**) SEM image and (**b**) EDS analysis of polished surface of 72 wt.% PiGs; (**c**,**d**) 3D reconstruction and surface CLSM images of 72 wt.% PiGs; (**e**) XRD patterns of APS, Isobam, PiGs, phosphors and glass powders.

Figure 5a presents SEM image of polished surface of 72 wt.% PiGs. There are a very limited number of pores in PiGs and the phosphors can be clearly distinguished from the glass matrix. It implied that PiGs had high compactness and phosphors were not eroded by the glass matrix. From Figure 5b, the analysis of elements in PiGs is obtained. The elements of Y, Al, and Ce were from phosphors, while the element of Si was from glass matrix. The most important point was that the content of element C was little which came from the measurement error. It meant that organic matter was completely excluded. As seen from Figure 5c,d, the distribution of phosphors is very uniform. This is one of the advantages of gel casting [31]. The presence of water promoted the mixing of the slurry. From Figure 5e, it is clearly observed that the characteristic peaks of Isobam, APS, and phosphors are not found, but the characteristic peaks of phosphors are observed in the PiGs. This not only proved again that the organic matter has been completely removed, but also showed that the phosphors were not destroyed during the sintering process. It reflected the reasonableness of the sintering process.

### 3.4. Optical Performances of PiGs

**Figure 6:**
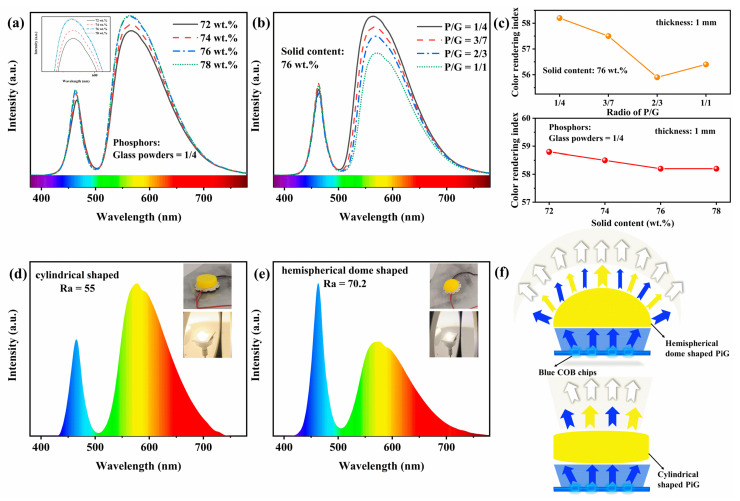
(**a**) The EL spectra of PiGs with different solid contents and (**b**) ratios of phosphors and glass powders; (**c**) Ra of PiGs with the thickness of 1 mm under different solid contents and ratios; (**d**,**e**) the EL spectra of cylindrical and hemispherical dome-shaped PiGs; (**f**) schematic diagram of cylindrical and hemispherical dome-shaped PiGs lit by blue COB chips.

Figure 6a,b illustrate the electroluminescence (EL) spectra of PiGs with different solid contents and ratios. Spectral intensity increased with increasing solid content in the same ratio. The intensity of yellow lighting increased with increasing solid content from 72 wt.% to 78 wt.% at the same ratio. The increase in density increased the effective conversion efficiency of phosphors to blue light [8]. When the solid content was remained at 76 wt.%, an increase of phosphors and a decrease of glass powders led to a reduction in the intensity of yellow intensity. It was mainly attributed to the weakening of transmittance. This was very understandable that the transmission rate was bound to decrease significantly when the phosphors increased and the glass powders decreased. Solid content and ratio of phosphors and glass powders have little effect on the color rendering index (CRI) of PiGs, as seen in Figure 6c. However, Figure 6d,f represent that the change in shape of PiGs is a huge improvement on CRI. The CRI of cylindrical and hemispherical dome-shaped PiGs were 55 and 70.2 respectively. After changing the shape from a cylinder to a dome, the CRI of PiGs was increased by around 27%. The cause of this resided in the change in shape. This resulted in changes in the transmissive capability of PiGs. It was also implied by the EL spectra shown in Figure 6d,e. Then, this would optimize the ratio between blue light and yellow light to produce a better quality of white light as displayed in Figure 6f.

### 3.5. Heat Dissipation Performances of PiGs

**Figure 7:**
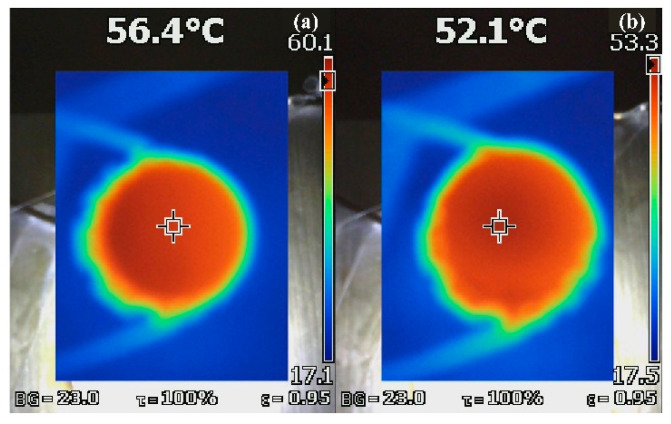
(**a**) Operating temperatures of hemispherical dome and (**b**) cylindrical-shaped PiGs under 10 W COB chips after 180 s.

Figure 7 describes the operating temperatures of hemispherical dome and cylindrical-shaped PiGs reaches 56.4 °C and 52.1 °C respectively under 10 W COB chips after 180 s. It was not difficult to find that the operating temperature of cylindrical-shaped PiGs was lower because heat dissipation effect of PiGs with larger surface area was better and the surface area of cylindrical-shaped PiGs was larger than hemispherical dome-shaped ones. In conclusions, the heat dissipation performance and optical performances of PiGs can be further improved by preparing suitable shapes by gel casting to make it suitable for high-power WLEDs lighting.

## 4. Conclusions

PiGs were prepared successfully by adopting gel casting with Isobam for the first time in this study. It was found that the Tg of glass powders, sintering rate, and the content of phosphors and glass powders could affect the preparation of PiGs. The Tg of glass powders must be higher than Ts of organics. A very slow sintering rate should be used in the sintering process, otherwise it was easy to cause deformation of PiGs. The optimum solid content was 76 wt.% by considering the rheology behavior of the slurry and the shrinkages of green bodies. When the solid content was more than 76 wt.%, the rheological behavior of slurry was poor and it was difficult to cast. When the solid content was less than 76 wt.%, the shrinkage rate was too high and the density was low. The most important point was that the CRI and heat dissipation effect of WLEDs could be significantly improved by changing the shape of PiGs. The CRI of PiGs could be increased by about 27% through changing the shape from cylinder to dome. Operating temperature could be effectively reduced by increasing the surface area. Therefore, the advantages of PiGs in high-power WLEDs lighting can be further expanded by preparing appropriately shaped PiGs.

## Figures and Tables

**Table 1:** Composition of samples with different ratios of phosphors and glass powders.

Sample	Solid Content (wt.%)	Glass Powders (g)	Phosphors (g)	Isobam (g)	APS (g)
Sample1	72	40	10	1.5	0.5
Sample2	74	40	10	1.5	0.5
Sample3	76	40	10	1.5	0.5
Sample4	78	40	10	1.5	0.5
Sample5	76	35	15	1.5	0.5
Sample6	76	30	20	1.5	0.5
Sample7	76	25	25	1.5	0.5

## Data Availability

Data will be made available from the corresponding authors on reasonable request.
